# Cannabinoid hyperemesis syndrome in the pregnant patient: clinical case and literature review

**DOI:** 10.1186/s12245-020-00311-y

**Published:** 2020-10-28

**Authors:** Julien Flament, Nathan Scius, Henri Thonon

**Affiliations:** Emergency Department, CHU UCL Namur, 1, rue Dr G. Therasse, 5530 Mont-Godinne, Yvoir, Belgium

**Keywords:** Cannabinoid hyperemesis syndrome, Pregnancy, Cannabis, Vomiting, Cyclical vomiting syndrome

## Abstract

**Background:**

Cannabis use is on the rise. Several cases of cannabinoid hyperemesis syndrome, secondary to chronic cannabis intoxication, have been described worldwide, but few cases have described this entity in pregnant women.

**Case presentation:**

We describe a 29-year-old pregnant patient that had consumed cannabis and experienced uncontrolled vomiting. The use of hot baths, the rapid improvement in symptoms, and results of complementary examinations suggested a diagnosis of cannabinoid hyperemesis syndrome. The patient could return home, and she continued her pregnancy and childbirth without peculiarities.

**Conclusion:**

Cannabinoid hyperemesis syndrome should be considered in the differential diagnosis of vomiting in pregnancy. Consumption of cannabis must be systematically included in the anamnesis. However, it seems to be somewhat unacceptable socially or medically. Consumption must be stopped to manage symptoms.

## Background

Cannabinoid hyperemesis syndrome is a recent clinical entity [1] involving nausea and vomiting in chronic cannabis users. The syndrome is also frequently associated with abdominal pain and compulsive showers or hot baths, which can attenuate the symptomatology [1, 2].

The diagnosis of cannabinoid hyperemesis syndrome is based on the exclusion of other causes, and the disappearance of symptoms once cannabis consumption is terminated [2]. The diagnosis and treatment of cannabinoid hyperemesis syndrome presents a challenge to the clinician. We describe a rare case of a pregnant woman that was likely to have cannabinoid syndrome. We will review the cases published to date and the current therapeutic options and discuss the peculiarity of pregnancy. Finally, we emphasize the notion that this syndrome should not be ignored, when assessing other causes of vomiting during pregnancy, such as hyperemesis gravidarum.

## Case presentation

A 29-year-old patient in her first pregnancy, at 29-week and 1-day gestation, visited the emergency room for uncontrollable vomiting and epigastric pain. She reported no health problems, but she smoked tobacco and cannabis. She had consumed 2 g per week for 6 years. She did not have any known allergies. She reported a decline in symptoms when taking a hot bath. The cardiopulmonary clinical examination was unremarkable. Abdominal palpation showed epigastric pain without guarding or rebound. The patient had a body temperature of 35.9 °C and displayed stable hemodynamics.

Further examinations revealed normal CBC but leukocytosis with WBC of 14.7 with a left shift. An abdominal ultrasound showed no detectable pathology. Obstetrically, she had a normal ultrasound, normal cardio-fetal monitoring.

She was treated with acetaminophen (1000 mg), tramadol (50 mg), metoclopramide (10 mg), and butylhyoscine bromide (10 mg). Based on the strong suspicion of cannabinoid hyperemesis syndrome and her clinical improvement in the emergency department, the details of this entity were explained to the patient, and she returned home.

The pregnancy was uncomplicated, and she gave birth at 39 weeks 6/7 to a healthy 2650-g newborn. She was discharged home with her infant uneventfully on postpartum day 3.

## Discussion

The classic clinical picture of cannabinoid hyperemesis syndrome is as follows: chronic, intensive use of cannabis, episodes of uncontrollable vomiting, and abdominal pain. A symptomatological and temporary improvement in symptoms is noted when taking a bath or hot shower. Finally, the syndrome ceases when the patient ceases cannabis use and resumes when they do not.

Cannabis use is increasing worldwide [3]. Accordingly, the number of published cases of cannabinoid hyperemesis syndrome has increased, and the pathology is of interest in various related specialties, including pediatrics [4] and forensic pathology [5]. Although the cause of the syndrome is being discussed continually and remains to be formally clarified, a few known factors are notable. First, Δ-9-tetrahydrocannabinol (THC) is a CB1 receptor agonist, and it is assumed that deregulation of the receptor could cause nausea.

More than half of pregnant women experience nausea and vomiting. Hyperemesis gravidarum, which affects only 0.3 to 1% of pregnant women, is defined as persistent vomiting, more than 5% weight loss, ketonuria, and electrolyte abnormalities (particularly hypokalemia). The physiopathology remains unclear, but it is linked to hormonal activity and the production of human chorionic gonadotropin [6, 7].

We conducted a literature search to identify clinical cases of cannabinoid hyperemesis syndrome in pregnant patients. We employed the search terms “Cannabinoid,” “Hyperemesis,” and “Pregnancy” in PubMed andGoogleScholar. We identified five clinical cases [8–12] and extracted their characteristics (detailed in the appendix tables) (Tables 1 and 2).

**Table 1:** Published cases of cannabinoid hyperemesis syndrome in pregnant women

Number	Reference	Age, y	Obstetric status	Gestation period, weeks; days	Nausea and vomiting + Cannabis use + hot shower/bath	Shower effect	Usual consumption (how long/how many times per week)	Antiemetic/effect	Other examinations	Delivery
1	Schmid et al., 2011 [8]	26	G2P0A1	10; ?/?	All 3	Beneficial	13 years/daily	Metoclopramide Chlorazine Dexamethasone Ondansetron Vitamin B6/ineffective	Urinary and blood tests	Uncomplicated
2	Alaniz et al., 2015 [9]	28	G5P3A1	30; 5/7	All 3	Beneficial	12 years/daily	Unspecified molecule/ineffective	Blood test + cerebral and abdominal magnetic resonance imaging	NICU for hypoxia. Hospital discharge on the second day in good condition
3	Andrews et al., 2015 [10]	24	G5P2A2	26; 2/7	All 3	Beneficial	8 years/several times per week	Treatment incompletely specified. Metoclopramide effectiveness associated with stopping cannabis use and a prescription of hot showers	Blood test Abdominal x-ray Abdominal and pelvic CT scan	37 wks 2270 g APGAR 9/9; No complications described
4	Manning Meurer et al., 2018 [11]	21	G1P0A0	6; ?/?	Shower/bath not described	?	?	Treatment: unspecified molecule/no description of efficacy	Blood test digestive endoscopy	Induced at 36 wks 6/7 for pre-eclampsia. 2430 g Fetal deceleration Shoulder dystocia APGAR 1/5 19 days in NICU Baby intracranial hemorrhage and spontaneous resorption. At 10 months of life, normal baby evolution
5	Kim et al., 2018 [12]	20	G7P0A6	14; 3/7	All 3	Beneficial	?	Ondansetron Famotidine Metoclopramide Ondansetron per os Promethazine IR All ineffective	Blood test	40 wks 1/7 3190 g APGAR 8/9 No complications described
6	This case	29	G1P0A0	29	All 3	Beneficial	6 years/daily	Metoclopramide/no effect	Blood test Abdominal and pelvic ultrasound	39 wks 6/7 2650 g No complications. Hospital discharge on the third day in good condition

*?* indicates no data available, *NICU* neonatal intensive care unit, *y* years, *wks* weeks, *g* grams

**Table 2:** Demographic characteristics

Characteristic	Average/median
Age, years	24.6/25
Pregnancy at diagnosis, weeks	19.1/20
Time since first consumption, years	9.75/10
Consumption at least several times a week, *n*	4/4
Beneficial showers/hot baths, n	6/6
Need for neonatal intensive care, n	2/6

Unlike studies in non-pregnant individuals [2], the low number of studies in pregnant patients makes it difficult to perform statistical analyses. On the other hand, there are similarities between non-pregnant and pregnant individuals. Most individuals with cannabinoid hyperemesis syndrome have consumed cannabis for years, mostly daily, and the symptoms tend to improve after taking a shower or hot bath. However, cannabinoid hyperemesis syndrome is particularly complex in pregnant women. Women that use cannabis during pregnancy are more likely to experience severe nausea than those that do not [13]. Nevertheless, and paradoxically, antiemetic effects have been attributed to cannabis, in both the general population [14] and pregnant women [15]. Some authors have recommended cannabis for treating hyperemesis gravidarum [16]. One hypothesis suggested that endocannabinoids played a role in the latter pathology. However, that hypothesis was refuted in a prospective study that did not find any changes in plasma endocannabinoid levels in patients with gravidarum hyperemesis [17].

Cannabis use is increasing among pregnant women [18]. However, it seems to be somewhat unacceptable socially or medically. This stigma might lead them to hide it when taking of a comprehensive patient history. This possibility was reinforced by the absence of a link between symptomatology and cannabis use [8]. The prevalence of cannabis use in parturition is likely to be generally underestimated [19]. Some patients diagnosed with classical nausea and vomiting during pregnancy or even gravidarum hyperemesis might actually have cannabinoid hyperemesis syndrome. These entities might be differentiated clinically by the sensitivity of hyperemesis gravidarum to certain antiemetic drugs [20, 21].

Potentially beneficial therapies for treating common cannabinoid hyperemesis syndrome, such as lorazepam [22] and haloperidol [23], are contraindicated in parturient women [24]. Capsaicin cream is not recommended in pregnant women. However, its usefulness could be discussed, in light of its supposed safety. Capsaicin treatment requires low dosages, and it does not cross the placental barrier [25]. Similarly, ondansetron [26] appears to have some efficacy. Although ondansetron is not recommended in the first trimester of pregnancy, its use might be permissible thereafter [24]. Metoclopramide, which can be used throughout pregnancy, has been used to treat cannabinoid hyperemesis syndrome and showed some efficacy.

This case study serves to remind physicians of the need to insist on recounting cannabis use when taking of the comprehensive patient history, particularly in parturient women with vomiting. Fortunately, the compulsive use of hot baths or showers seems specific to this clinical entity [2] and can help discriminate cannabinoid hyperemesis syndrome from other diseases.

There is a need for prospective studies that aim to determine the proportion of this syndrome among a parturient sample with vomiting and characterize potential differences in this subpopulation.

Randomized, controlled therapeutic trials should also be conducted to find a functional treatment for symptoms. On the one hand, an effective treatment could obviate the potential consequences of drugs of equivocal safety. On the other hand, an effective treatment is needed to avoid serious consequences of uncontrollable vomiting, which range from pneumomediastinum [27] to death [5] and acute renal insufficiency [28].

## Data Availability

Please contact author for data requests.

## Abbreviations

CBC: Complete blood count
WBC: White blood cell
